# Enhancing cancer care through digital social care referrals: insights from the ConnectedNest pilot study

**DOI:** 10.1007/s00520-025-09523-5

**Published:** 2025-06-05

**Authors:** David Haynes, Eric Trempe, Allison Iwan, Eduardo Osegueda, Courtney Sarkin, Dori Cross, Abbie Begnaud, Kris Newcomer, Helen M. Parsons, Pinar Karaca Mandic

**Affiliations:** 1 Institute for Health Informatics, University of Minnesota, Minneapolis, MN USA; 2 Xanthos Health, Inc, Dudley Avenue, Saint Paul, MN USA; 3 Computer Science and Engineering, University of Minnesota, Minneapolis, MN USA; 4 School of Public Health, University of Minnesota, Minneapolis, MN USA; 5 Medical School, University of Minnesota, Minneapolis, MN USA; 6 Minnesota Cancer Alliance, Minneapolis, MN USA; 7 Carlson School of Management, University of Minnesota, Minneapolis, MN USA

**Keywords:** Health-related social needs, Community-based organizations, Digital social care referral platform

## Abstract

**Purpose:**

Over 8 million patients experiencing cancer face health-related social needs (HRSN) challenges, such as food, housing, and financial insecurity, that directly impact their health outcomes. While patients may participate in the screening of unmet social needs with their healthcare provider, it is not well understood if patients will self-screen and act on referrals to community services. Therefore, we conducted a mixed-methods study in which we pilot-tested a patient-centered oncology-focused digital social care referral platform, ConnectedNest.

**Methods:**

We recruited 13 cancer-focused community-based organizations (CBOs) and 41 individuals (e.g., patients, survivors, and caregivers) affected by cancer to participate in the study. Individuals used the application on their phones for 3 months. They completed a social needs assessment and were provided with a personalized list of local CBO programs that could address these unmet needs. Quantitative analyses described participant HRSN and connections to referred programs. User experience surveys were collected over 30 and 60 days to understand their experience. Individuals were also invited to participate in an a semi-structured interview to understand barriers and facilitators for social care referrals.

**Results:**

Participants reported needs across 14 categories, with an average of 4.5 HRSN per participants. The top 3 reported needs were physical activity, financial strain, and mental health. Using our social care referral platform, approximately 39% of participants were able to connect with local CBOs. Three topic areas emerged from the participant interviews, highlighting that personal motivation, the participant's role, and the interest in services affected the participant's use of the technology.

**Conclusion:**

This study demonstrates the potential for digital social care referral platforms to bridge individuals affected by cancer with vital CBO resources to address HRSNs.

**Supplementary Information:**

The online version contains supplementary material available at 10.1007/s00520-025-09523-5.

## Introduction

There are approximately 16.9 million cancer survivors in the USA [1]. There is a growing body of literature demonstrating that many cancer outcomes are affected by individual-level health-related social needs (HRSN) [2]. These concerns, which are part of the Social Determinants of Health (SDoH), are challenges that exacerbate access to health and health care, including transportation, language proficiency, and financial hardship [3]. Zettler and colleagues reported that approximately 8 million patients (half of all survivors) experience some unmet social care need [4]. SDoH are population measures of socioeconomic conditions, and HRSN are individual measures of socioeconomic conditions [5]. Patients experiencing cancer are at particular risk for the adverse effects of HRSN, given the dramatic growth in the financial burden of cancer over the past decades [6, 7]. Cancer is the second most expensive chronic disease [4, 8] with the annual cost of many new cancer drugs being $100,000 or higher [7]. Additionally, health insurers are increasingly shifting the cost of cancer care to patients who are experiencing growing out-of-pocket costs [4, 9]. Some studies have reported financial toxicity (e.g., debt, bankruptcy, delaying care because of cost) in more than 50% of cancer survivors [10]. Additionally, patients who experience cancer are more likely to experience food insecurity compared to the general population [11]. Therefore, individuals who experience cancer need help connecting to supportive resources.

Tools like the Distress Thermometer [12, 13] and the Problem List [14] have been in use since the early 2000 s to identify psychosocial factors and HRSNs that affect patients [15]. However, the problem is not the lack of screening tools. The literature suggests that patients and healthcare teams are overwhelmed by the complexity of unmet HRSNs [16–18]. Integrating distress screening into routine care is complex [19–21] and requires novel technology to seamlessly connect patients and healthcare teams to community-based organizations (CBOs). Therefore, we developed and tested a new model of health information technology that uses a patient-centered framework designed for patients experiencing cancer [21, 22]. The goal of this study was to test the feasibility and acceptability of a digital social care referral platform for connecting individuals experiencing cancer with social care services provided by CBOs.

## Methods

ConnectedNest is a digital social care referral platform that helps patients, communities, and healthcare systems conduct social care referrals in a closed-loop solution. ConnectedNest consists of three interfaces customized for patients (EmpowerNest), CBOs (CommunityNest), and healthcare teams (EngageNest). EmpowerNest is a mobile app that allows the patient to self-screen, self-refer, and manage (add/remove/change) their own HRSN. It does not require face-to-face interactions with providers, potentially reducing stigma. CommunityNest is a web portal that allows CBOs to create and maintain their programs. CBOs have full control of their programs, indicating the start and end date, the capacity of each program, and defining eligibility criteria. EngageNest is an electronic health record (EHR) app supported by Substitutable Medical Applications and Reusable Technologies (SMART) on Fast Healthcare Interoperability Resources (FHIR). Clinicians and social workers can view available services, refer patients to CBOs without sharing patient information, and track each patient’s progress. Further details about the platform can be found in Parsons et al. [22] and Haynes et al. [23] The EHR interface was not used in this study.

To test ConnectedNest, we conducted a 3-month pilot study with 41 cancer survivors and caregivers and 13 CBOs. Both survivors and CBOs were recruited from the Minnesota Cancer Alliance, which is supported by the Minnesota Department of Health through the Comprehensive Cancer Control Program from the Centers for Disease Control. This research was determined to be non-human subjects by the University of Minnesota Institutional Review Board.

### Cancer-focused CBOs

We recruited 13 CBOs with missions focused on serving patients, survivors, and caregivers affected by cancer to participate in the pilot study. While it was a convenience sample, we selectively recruited CBOs to curate a diverse platform in terms of 1) the services each CBO offers (e.g., food, financial, physical fitness); 2) the specific types of cancer supported (e.g., breast cancer, ovarian cancer, colon cancer, etc.); and 3) their target audiences (e.g., patient, survivor, and/or caregiver) CBO staff participated in monthly meetings with the University of Minnesota study team. These meetings allowed CBOs to inform design decisions and discuss barriers and facilitators of implementing the technology.

### Study recruitment and data collection

Study participants were recruited through a continuous convenience sample of the 13 CBOs [24]. They facilitated recruitment through their advocacy and outreach events and promoted enrollment through their existing client networks. Organizations disseminated flyers in public places that allowed members to register to use the application. Participant recruitment began on August 1, 2023, and ended on November 30, 2023. Organizations also employed a variety of approaches to recruitment, including targeted recruitment and one-on-one engagements, dissemination via social media or mailing lists, and recruitment during existing organizational events (e.g., support groups and fundraiser activities). All community members who consented agreed to participate in the study for 3 months. Our final verified study population was 41 participants.

Participants used the app on their mobile device and were directed in the app to complete a set of modules, which included a demographic assessment for the creation of a profile and the Accountable Health Communities survey by the Centers for Medicare and Medicaid Services [25, 26]. After registration and verification, participants were able to use the app to connect with organizations that could provide them with services. Participants were then sent a secure message to participate in an acceptability survey after 30 and 60 days. All enrolled participants who completed at least one survey (*n* = 28) were asked upon completion of the study if they would participate in an interview. Attempts were made via telephone to reach the initially non-responsive participants. Participants were emailed two times, and up to three phone calls were attempted for all participants. Those who agreed to participate were interviewed for approximately 30 min and received $25 via gift card for their time.

### Quantitative analysis of participant use of the social care referral platform

We used the outcomes from Addressing SDoH in Systems (OASIS) framework to guide our data collection process [27]. OASIS organizes data collection for process measures around unmet social needs and health outcomes. Two datasets were collected during this pilot study. The first dataset was a compilation of information obtained from participant responses and app usage information within ConnectedNest. ConnectedNest collected both the participants’ demographic information and identified HRSN. ConnectedNest also monitored actions taken by participants on the platform and stored this information within a PostgreSQL database [28]. For example, ConnectedNest tracked participant-identified needs, participants requesting a connection to a program, and the status of a social referral. The second dataset was the acceptability survey distributed via email through REDCap [29] at 30 and 60 days. We report descriptive statistics of the dataset obtained through ConnectedNest and the self-reported survey results.

#### ConnectedNest demographics

Participant demographic information was collected for the analytical purposes of this study and for ConnectedNest’s matching algorithm. Patients self-report their gender, race/ethnicity, age, ZIP code, and additional characteristics. ConnectedNest used an algorithm to rank and match patients to available CBO programs.

#### HRSN assessment

The Accountable Health Communities assessment was modified to align the questions with the programs provided by the CBOs. Our assessment included the majority (22) of the original 26 items. Four questions related to substance abuse were removed as CBOs did not address this concern, and we added two questions about financial education and the need for legal assistance, which resulted in a 24-item assessment (Appendix 1).

#### Acceptability survey

We developed an acceptability survey based on the System Usability Scale [30]. The 10-item assessment had a mixture of multiple-choice (7) and open-ended questions (4), all of which could be skipped (Appendix 2). The questions focused primarily on the app's acceptability and usability while also allowing participants to describe potential challenges.

### Qualitative analysis of patient experience and motivation

From November 2023 to February 2024, we contacted participants who completed at least one HRSN screening assessment via email for an interview (Appendix 3). Attempts were made via telephone to reach the initial non-responsive participants. Participants were emailed two times, and up to three phone calls were attempted for all participants. Those who agreed to participate were interviewed for approximately 30 min and received $25 via gift card for their time. Interviews were conducted using Zoom, which was subsequently transcribed by Zoom and/or using the rev.com AI transcription service. Three study team members then independently coded transcripts to develop themes for each of the key topics discussed above. Our analysis approach used grounded theory [31, 32]

## Results

**Fig. 1:**
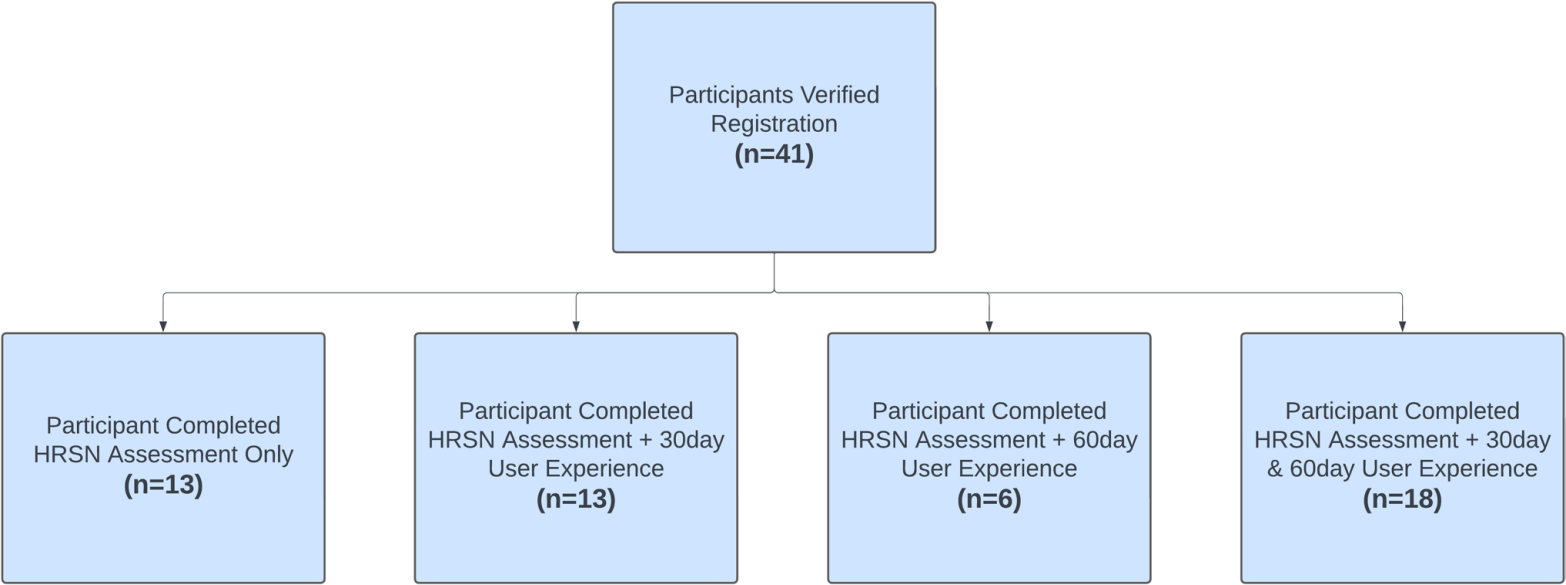
CONSORT diagram detailing completion of assessment activities by participants

The 41 participants enrolled in this study can be subdivided into four different groups based on their level of participation (Fig. 1). Table 1 shows the self-reported demographic information from participants when they created their profiles within the application. These results show that the two subpopulations within our study are similar. There are no substantial differences between participants who only completed the HRSN assessment and those who completed the HRSN assessment and at least one additional usability and experience survey (Table 1). The recruited sample reflects the population in Minnesota that is experiencing cancer.

**Table 1:** Participant population description

	Participant completed HRSN assessment only (*n* = 13)	Participant completed HRSN assessment and at least one additional assessment survey (*n* = 28)
Age		
20–39	15% (2)	18% (5)
40–59	62% (8)	50% (14)
60–80	23% (3)	32% (9)
Race/ethnicity		
Black or African American	8% (1)	7% (2)
Hispanic or Latino	0% (0)	4% (1)
White	77% (10)	79% (22)
Did not answer	15% (2)	11% (3)
Gender		
Female	62% (8)	79% (22)
Male	38% (5)	18% (5)
Did not answer	0% (0)	4% (1)
Disability status		
Disabled	8% (1)	14% (4)
Not disabled	85% (11)	75% (21)
Did not answer	8% (1)	11% (3)
Veteran status		
Veteran	8% (1)	4% (1)
Nonveteran	85% (11)	93% (26)
Did not answer	8% (1)	4% (1)

**Fig. 2:**
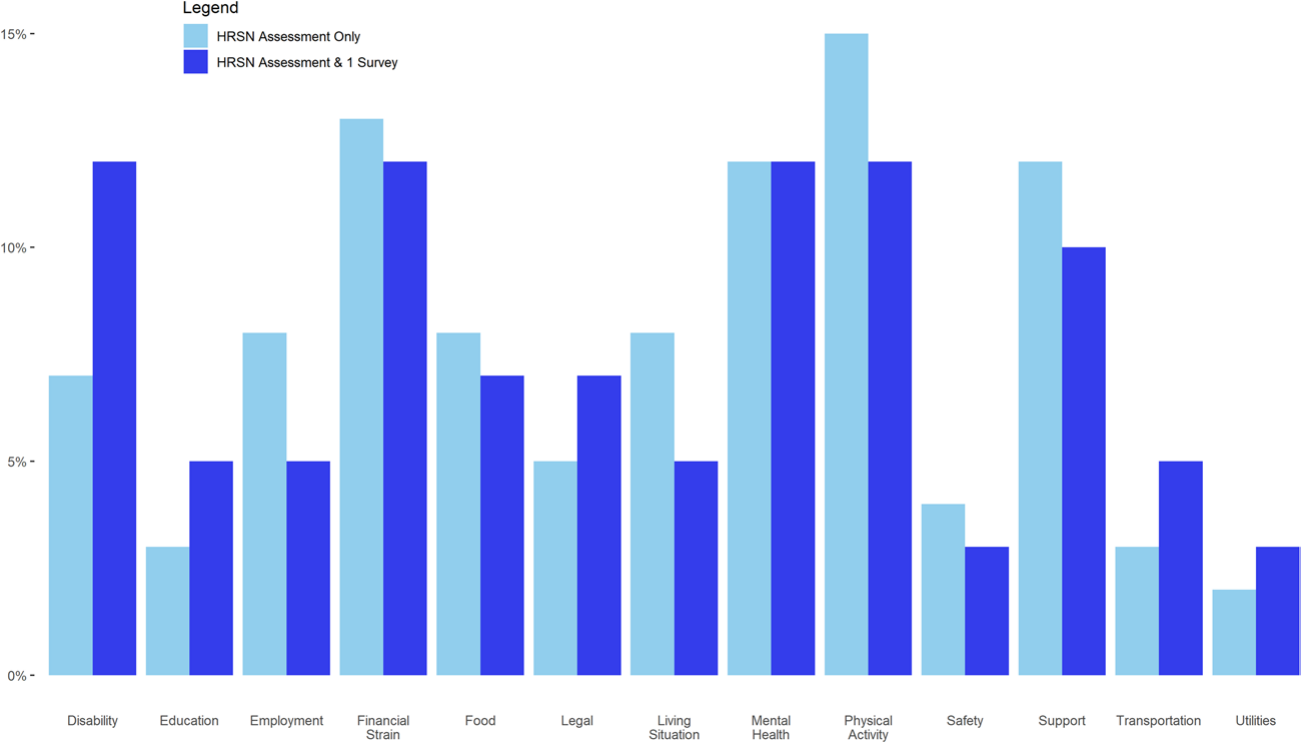
Percentage of participant needs by type of assessment completed

Overall, the HRSN of the two groups was similar (Fig. 2). Physical activity, financial strain, and mental health were the three HRSNs that individuals identified the most. Utilities and transportation were the HRSNs that the fewest members identified. The one irregularity we identified was in the disability category. Members who did not complete the HRSN assessment had a smaller percentage of disability needs (7.6%) than those who completed the HRSN (14.3%).

### Health-related social needs identified through ConnectedNest

**Fig. 3:**
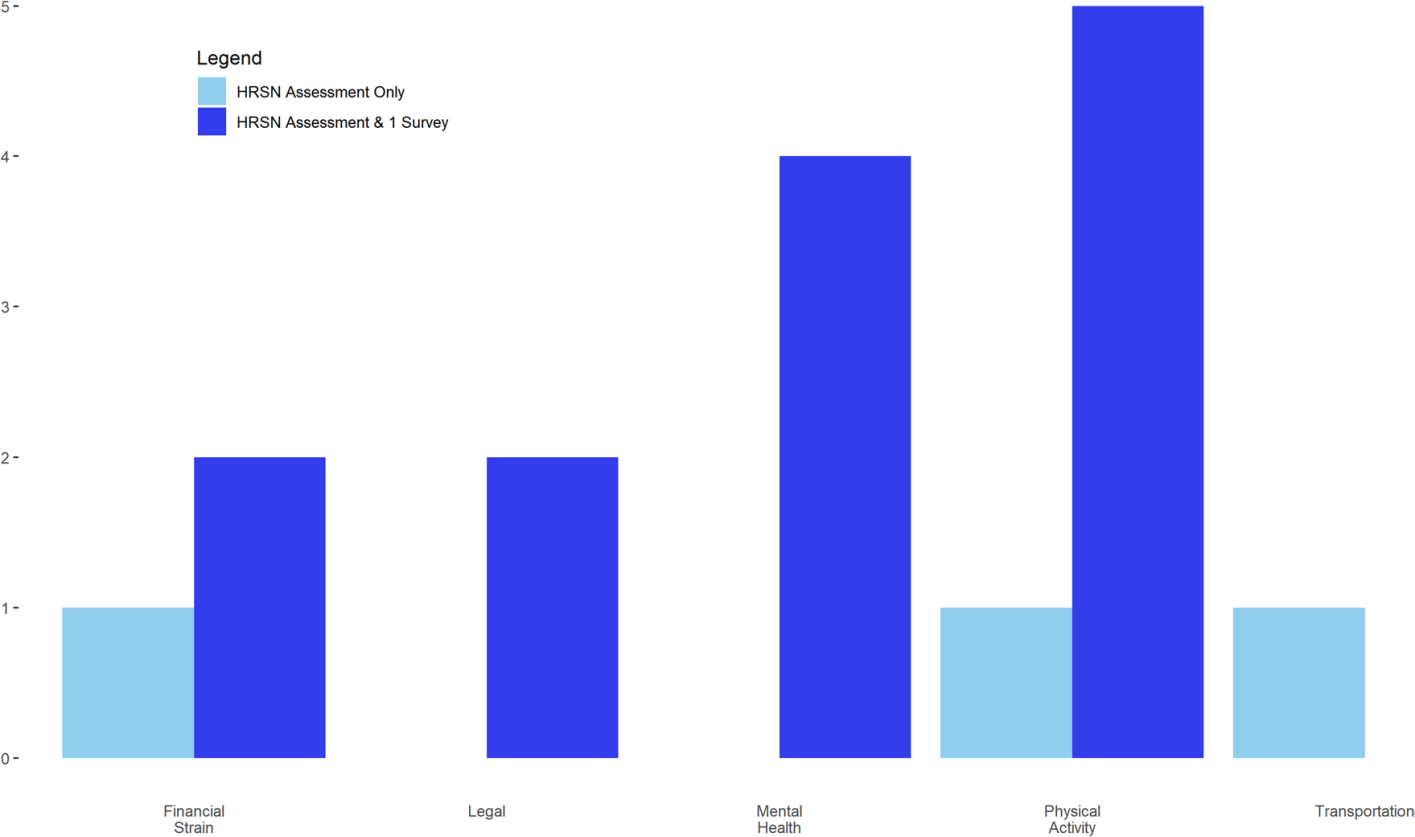
Number of health-related social need connections made

Figure 3 reports the number of HRSN connections to CBOs. A program connection was defined as a patient choosing an unmet need within the application, reviewing the program description, and then choosing to connect with that organization. The connection was completed when the organization reviewed and chose to accept the patient into the program. Our results showed that 39% (*n* = 16) of participants were able to connect to services. Even participants who only completed the HRSN assessment were connected to programs. Physical activity was the program with the most connections. This was a specialized program designed by licensed personal trainers for individuals with cancer.

Table 2 reports that participants identified 4.5 HRSNs on average. While participants had multiple unmet needs, they did not always make a connection. There were 21 total connections made between participants and CBOs on the platform. Approximately 1 in 4 users in the HSRN-only group attempted to connect with an organization, whereas in the HRSN and at least one survey, the rate was higher at about 1 in 2. The technology also facilitated quick connections between participants and organizations.

**Table 2:** Characteristics of ConnectedNest organization connections

	Participant completed HRSN assessment only	Participant completed HRSN assessment and at least one additional assessment survey
Total number of connections made	3.0	18.0
Average needs per user	4.46	4.64
Average connections per user	0.23	0.45
Average days per connection request	0.33 day	0.50 days (**4.91 days)
Longest wait	1 day	1.5 days (**39 days)

**Denotes outlier of 39 days included

### Survey results of participants’ experience with ConnectedNest

Participants who completed an acceptability survey (*n* = 28) self-reported 11 connections with organizations. This is lower than the 16 connections collected by ConnectedNest. All of these self-reported connections were reported during the first assessment, which was 30 days after using the technology. Those who made connections were evenly distributed between connecting with a new CBO and connecting with an organization with which they had an existing relationship. Participants connected with a new organization they previously did not know about, and they also connected with organizations they knew. Among participants who did not make a connection, lack of unmet needs and infrequent use of the platform were their reasons.

Over 80% of participants identified that they enjoyed using the technology. We also asked participants about the features that they liked best. The top two responses were being able to find services (36%) and having the application on their device (36%). The HRSN assessment was also rated very highly, with 32% of the participants enjoying this feature. The fourth most popular item was the descriptions of the programs. The category with the fewest responses was the referral updates, which are notifications (emails) that the participants would have received to inform them of progress made in completing the social care referral.

### Key themes identified from participant interviews after using ConnectedNnest

We completed 14 interviews with study participants. The majority were female (*n* = 13), one was male, nine of those interviewed were aged 40–59, and five were aged 60–80. Additionally, 12 of the participants self-identified as White. Three key topics were identified through our analysis of the interviews (Table 3).

**Table 3:** Topics and themes from patient interviews

Topic	Themes	Exemplary quotes
Topic 1. Motivation to participate in the ConnectedNest pilot study	Technology facilitated awareness, knowledge, and access to resources	Quote 1: “I feel like there, it's really hard to find the resources. And also, I mean partially because I started this journey through COVID. And so many of the resources that were once available are no longer available or were on pause at that time.”
		Quote 2: “…the platform allows a person—allows you—to go back and look, and maybe review something else that you might have been too proud to look for”
		Quote 3: “This could really revolutionize the way that people are cared for during their treatment. And it's a big deal and having peace of mind while you're going through treatment… you shouldn't have to spend a lot of extra time on is really huge to your recovery and healing
		Quote 4: “I was looking for resources and I tend to be someone who likes things that are online versus going to things in person. Um, uh, or calling. I don't like to call and ask questions, so I'd rather just go online and see what's available and send off an email or a text or something.”
	Caregivers are vital users	Quote 1. “I went at it as a friend, thinking I have a friend with cancer who needs help with caregiving.”
		Quote 2: “I did share some of the resources that I found on the platform with other people who are currently going through their own journey of cancer”
Topic 2. Participants’ cancer journey impacted their involvement	The platform needs to serve individuals differently across stages of the cancer journey	Quote 1: “So when I started, you know, working with this ConnectedNest program, it was. I was already finished with my treatment, and so most of the programs then don't apply anymore.”
		Quote 2: “I have maybe more time to have reached out, where someone who's very new to cancer, these people would be totally new to them. … So it's not new to me, but I can see how it'd be new to someone else.”
		Quote 3: “I didn't, because where I'm currently at in my care, there isn't anything that has something I would be able to benefit from.”
Topic 3. Participants were interested in a wider range of service offerings	Sometimes resources were available, but it was unclear to users what range of services each CBO could offer	Quote 1: “I was also looking for direct coaching for cancer patients. Again, relative to things like complementary care relative to things, like emotions relative to things like to stress, to resilience, to communication with loved ones. Just that type of coaching sort of like chronic disease coaching. But I didn't see that. And I was looking for that as well.”
		Quote 2: “I saw a lot of stuff for probably people who like need financial assistance since, and more of the government assistance stuff. But I didn't see stuff for more, so people who have, you know, medical insurance, are looking more for support groups and more like wellness and well-being stuff.”
	Users expected to see more resources	Quote 1: “at the times that I did the site…There weren't a whole lot of resources.”
		Quote 2’ “I don't think there was educational things in there, or at least it didn't fit what I was looking for…”
		Quote 3: “It's like I was looking for things like from the center, for spirituality and healing like their services, like massage, like Tai Chi, like that kind of support for cancer patients.”

The first topic encompassed participant motivation. Participants were motivated to participate due to their personal struggles connecting to resources. Quotes suggested that the technology would transform their approach to finding resources, which would reduce their stress and help them find new resources. We also learned that participants would utilize the technology differently to connect with organizations; some would use the in-app connection capabilities, while others would connect directly on their own. The importance of caregivers and the role they might play in this process was also highlighted. Participants suggested that ConnectedNest could be as beneficial for caregivers as for the patients themselves.

The second theme we identified was the participants’ status in their cancer journey, specifically that ConnectedNest could be used to serve individuals differently across multiple stages of the cancer journey. Since participants were recruited from cancer-focused CBOs, many stated that they were beyond the social need phase of their cancer journey. Participants had experienced looking for these programs (theme 1—motivation), but had been connected to some resources that addressed some of these concerns.

The third theme discussed how the resources available within the platform aligned with participants’ perceptions. Overall, participants wanted more programs and resources within the platform. The resources available to cancer patients vary widely, based on the cancer type and stage in the cancer journey, and participants wanted access to all of these. Suggested resources could include cancer coaching, complementary medicine, and programs for individuals with or without insurance.

## Discussion

The results of our study suggest that a patient-centered social care referral platform can be useful for connecting patients experiencing cancer to social resources in their community. Our study reported that 39% of patients were able to connect to resources. This reported rate is higher than the rates of patients who have participated in SDoH screening and connected with their referrals in other studies [16, 33, 34]. Friedman and Banegas [16] have the highest reported rate of program utilization at 23%, whereas Schickedanz et al. and Lindau et al. [33, 34] reported rates of 10 and 11%, respectively. ConnectedNest also facilitated connections across a wide range of program services. The 13 CBOs created 53 programs that spanned 11 need types (i.e., disability, education, employment, financial, food, legal, housing, mental health, physical activity, support, and transportation). A full description of the CBO organizations is in Appendix 4. The program category that had the most programs (*n* = 18) was support, while physical activity had the most connections (Fig. 3). Future research should explore why connections were not made to these programs.

Our pilot also provided valuable insights that could address knowledge gaps within the social care referral space. Currently, healthcare system staff are unaware if a patient has connected with and/or received resources from a CBOs and relies on patient self-report. Our study used both self-reported (*n* = 11) and technology-confirmed (*n* = 16) connections to CBOs. The technology-confirmed referrals in our study were reported at a higher rate, which is likely to have reduced recall bias. Additionally, the technology-confirmed referrals provided a deeper understanding of the steps involved in this process. A connection required that the patient choose to connect with a CBO program and that the CBO organization reviewed and confirmed the eligibility of the participant. Therefore, the implementation of a technology-driven referral system could address a critical data gap in social referrals. ConnectedNest can provide social care referral status information to connected healthcare team members.

We also report that three individuals who did not complete our acceptability assessments were still able to connect to resources. This suggests that the platform’s tailored recommendations were acceptable for participants. This addresses an existing challenge within the social care referral literature, which is that there is a need for highly specific referrals [35, 36]. While not everyone connected to resources, this study demonstrates that a tailored list of resources could be beneficial for patients looking for resources.

The results from our patient interviews align with the literature, suggesting that many patients have unmet social needs. Much of the literature has focused on the implementation of screening for distress or unmet social needs within oncology practices. A limited amount of research has examined when patients should be screened, when re-screening should occur, and has conducted longitudinal analyses of this data [37, 38]. Our results indicate that patient unmet needs change based on their cancer journey (Table 3—topic 2). Therefore, resources need to be characterized so that they can be given to patients at specific time points in their cancer journey.

Our last theme identified a lack of specific resources in the app. This lack of resources in social care referrals has been echoed in the literature mostly from the clinical side [17, 33, 39], [40]. In which providers state that they lack the resources to connect patients to resources effectively. Our study reported similar findings in that patients reported looking for resources and not finding them. This could be a result of only 13 CBOs participating in the study, or it could also be related to how the platform organized the resources. The lack of resources on the patient or clinical side identifies a more general problem. Patients have a number of unmet needs and are looking for specific resources. To address this problem, organizations will need to have a larger directory of resources and carefully tailor recommendations for patients.

## Limitations

We conducted a small 3-month pilot study in Minneapolis and St. Paul, MN, and it is likely that these results would not be generalizable to larger populations and different communities. Future work should focus on increasing the sample size of the study and implementing the app in different communities. Additionally, there were technical limitations during the pilot that prevented some participants from being able to access resources. This is likely to have reduced our total number of people connected to resources. The results from this study, showed that all connections were made within 30 days of starting the study. This could be due to our recruited participants being at various stages of their cancer journey, which is likely to affect their interest in social resources. Another limitation of the study was that we only recruited English-speaking participants and future research should include non-English speakers.

## Conclusion

This study demonstrates the feasibility of ConnectedNest, a digital social care referral platform, to connect patients experiencing cancer to CBOs and their resources. This work is currently focused primarily on the patients and the CBOs and future work is needed in additional areas. We conducted monthly meetings with CBOs who participated in the study and learned about the barriers that CBOs face when responding to participants. Understanding the roles of CBOs within the social care referral process is a major gap in the existing literature that we will continue to explore. Another area of future research is extending it to support caregivers. Future research will need to partner and recruit patients from healthcare systems to determine to what extent patients who are in active treatment would benefit from connection to resources. This will allow us to understand if these additional supports could improve treatment adherence and patient quality of life. Another area of future work would be the piloting of the technology with a health system and integrating the EMR interface. This would allow for a better understanding of the workflow of multi-disciplinary oncology teams and adapting the technology to support this diverse stakeholder group.

## Supplementary Information

Below is the link to the electronic supplementary material.

ESM 1 (DOCX 135 KB)

## Data Availability

No datasets were generated or analysed during the current study.
