# Supplementary material for: Enhancing cancer care through digital social care referrals: insights from the ConnectedNest pilot study
Source: Support Care Cancer. 2025 Jun 5;33(7):539. doi: 10.1007/s00520-025-09523-5 (PMC12141133; doi:10.1007/s00520-025-09523-5)
Supplement: Supplementary file 1 — (DOCX 135 KB) [file 520_2025_9523_MOESM1_ESM.docx]

Appendix

# Part 1: SDoH Assessment Questions

Living Situation

1. What is your living situation today?

I have a steady place to live

I have a place to live but I am worried about losing it in the future

I currently do not have a steady place to live

1. Think about the place you live. Do you have problems with any of the following? Choose all that apply

Water leaks

Pests

Mold

Lack of heat

Oven not working

Smoke detectors not working

Lead paint/pipes

Food

1. Within the past 12 months, you worried that your food would run out before you got money to buy more.

Often

Sometimes

Never

1. Within the past 12 months, the food you bought just didn't last and you didn't have money to get more.

Often

Sometimes

Never

Transportation

1. In the past 12 months, has lack of reliable transportation kept you from medical appointments, meetings, work or from getting things needed for daily living?

Yes

No

Utilities

1. In the past 12 months has the electric, gas, oil, or water company threatened to shut off services in your home?

Yes

No

Already shut off

Safety

1. How often does anyone, including family and friends, physically hurt you?

Never

Rarely

Sometimes

Fairly Often

Frequently

1. How often does anyone, including family and friends, insult or talk down to you?

Never

Rarely

Sometimes

Fairly often

Frequently

1. How often does anyone, including family and friends, threaten you with harm?

Never

Rarely

Sometimes

Fairly often

Frequently

1. How often does anyone, including family and friends, scream or curse at you?

Never

Rarely

Sometimes

Fairly often

Frequently

Financial Strain

1. How hard is it for you to pay for the very basics like food, housing, medical care, and heating? Very hard

Somewhat hard

Not hard at all

Financial Education

1. Do you want expert information on, or assistance with, financial planning, debt management, financial budgeting, assigning beneficiaries, planning for retirement, or legacy planning?

Yes, information and assistance

Yes, information only

No, I do not need or want help

Employment

1. Do you want help finding or keeping work or a job?

Yes, help finding work

Yes, help keeping work

No, I do not need or want help

Patient, Family, and Community Support

1. If for any reason you need help with day-to-day activities such as bathing, preparing meals, shopping, managing finances, etc., do you get the help you need?

I don’t need any help

I get all the help I need

I could use a little more help

I need a lot more help

1. How often do you feel lonely or isolated from those around you?

Never

Rarely

Sometimes

Often

Always

1. Do you speak a language other than English at home?

Yes

No

Education

1. Do you want help with school or training? For example, starting or completing job training or getting a high school diploma, GED or equivalent.

Yes

No

Physical Activity

1. In the last 30 days, other than the activities you did for work, on average, how many days per week did you engage in moderate exercise (like walking fast, running, jogging, dancing, swimming, biking, or other similar activities)?

0

1

2

3

4

5

6

7

1. On average, how many minutes did you usually spend exercising at this level on one of those days?

0

10

20

30

40

50

60

90

120

150 or greater

Mental Health

1. Over the past 2 weeks, how often have you been bothered by little interest or pleasure in doing things?

Not at all

Several days

More than half the days

Nearly every day

1. Over the past 2 weeks, how often have you been bothered by feeling down, depressed, or hopeless?

Not at all

Several days

More than half the days

Nearly every day

1. Stress means a situation in which a person feels tense, restless, nervous, or anxious, or is unable to sleep at night because his or her mind is troubled all the time. Do you feel this kind of stress these days?

Not at all

A little bit

Somewhat

Quite a bit

Very much

Disabilities

1. Because of a physical, mental, or emotional condition, do you have serious difficulty concentrating, remembering, or making decisions?

Yes

No

1. Because of a physical, mental, or emotional condition, do you have difficulty doing errands alone such as visiting a doctor's office or shopping?

Yes

No

Legal Services

1. Do you want to speak to an attorney regarding a cancer-related issue with your employment, estate planning, Social Security Disability Insurance-SSDI, or health insurance?

Yes

No

# Part 2: ConnectedNest app User Experience

It has been approximately one month since you registered with the ConnectedNest app and completed an assessment which is used to connect you to community-based organizations (CBOs).

Please complete the survey below to provide us with information about your experience that can help us improve it. Thank you!

start date/time

What do you like about the ConnectedNest app?
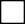
 Ability to find services that I need


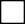
 Descriptions of the programs


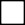
 Ability to connect with them on my device
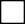
 Email notifications on referral status change
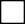
 Needs Assessment


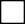
 Other

What other features do you like?

How easy or difficult has the ConnectedNest app been very easy to use? easy

neutral


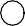

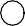

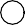

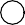

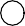


somewhat difficult difficult

Please briefly describe your experience here.

How has your overall experience been using the I fully enjoy using the app.

ConnectedNest app? I somewhat enjoy using the app.


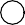

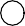

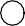


I do not enjoy using the app.

Why did you pick that rating?

Have you encountered any problems using the app? no

a few several


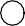

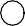

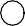


What issues were experienced?
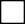
 Unable to find the services that I need
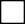
 Programs don't have good descriptions
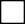
 Can't connect with any programs


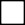
 The Assessment did not address/identify my needs
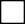
 Software/programming issues


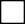
 Trouble navigating through/around the app
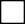
 Other

What other issues were experienced?


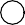

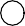


How often do you log into the app? I have only logged in once At least once a week Every day


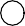

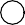

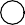

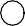


Whenever I receive an email notification about a program

What might make you more likely to continue using the app or using it more frequently?

How often do you search for services in the app? I haven't searched for services

At least once a week Every day


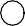

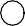

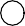

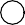


Other

Describe more fully how often you use the ConnectedNest app to search for services.

Through the ConnectedNest app, have you been able to Yes connect with one or more community-based organizations No (CBOs)?

Please indicate whether the CBO was one you already
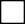
 I connected with a CBO I already work with. worked with or whether this was a new connection.
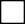
 I connected with a new CBO.


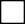
 Other


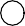

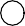


Mark all that apply.

Please explain your 'other' connection to this/these CBOs.

Have you received any services from a new organization Yes that you sent a request from within ConnectedNest? No

Please describe this experience.

Please indicate why you have not been able to connect
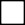
 I do not have any needs at this time. with a CBO.
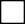
 The app did not match me to a CBO.


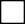
 I was not eligible for the CBO to which I was referred.


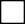
 The CBO has not yet responded.


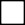
 I have not checked the app for a match.
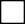
 I have not used the app since registering.
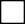
 Other

Please explain why there has not been a connection with a CBO.

Are there any additional features in the ConnectedNest app that you would like to see?

Please add any other comments about your experience using the ConnectedNest app over the past month.

stop date/time

Thank you for completing the survey. Click on the 'SUBMIT' button below.

If you have any additional comments or questions, please contact the ConnectedNest team at [ConnectedNest@umn.edu.](mailto:ConnectedNest@umn.edu)

As a token of appreciation for completing this survey, $25 will be added to your ClinCard within 7-10 days.

# Part 3: Participant Interview Guide

Hello, my name is [______], and I am from the University of Minnesota.

Thank you for your willingness to share your thoughts as we develop this new platform that you’ve been using called ConnectedNest, which is designed to support access to social and community services. When we say social and community services, we mean any types of non-medical services and supports that are needed or you would have wanted to connect with after a cancer diagnosis. We will ask how the connection with and use of these services can be made easier and what barriers you encountered. We want to make sure that any new technology is useful. There are no right or wrong answers, please share your honest thoughts and opinions coming from your own, unique experiences. Remember, this is a protected, safe space; anything said here is kept confidential.

This interview should take about 30 minutes of your time.

We are audio recording this discussion so we don’t miss anything. The only people who will ever hear this recording are study staff. The recording will be erased when the study is over.

Your participation is completely voluntary and has no impact on your health care. If you want or need to leave at any point, you may do that. We will follow-up with you to check-in but that’s it.

Please speak freely and from your experience.

What questions do you have?

Interview Questions:

**Motivation and Usage:**

1. We’re interested in understanding how useful ConnectedNest is. How did you first become connected with the platform?
   1. How, if at all, did this platform affect your relationship with the organization(s)?
2. What motivated you to sign up for the ConnectedNest platform pilot?
3. Thinking about your experience with the platform, how often would you say you logged into the platform?
   1. What might make you more likely to continue using the app or using it more frequently?
4. How often would you say you searched for services on the platform?
   1. [If no search for services]: Please tell me a little more about that.
5. Do you think a platform like this helped you learn about resources in your community?
   1. [If platform helped them learn about resources]: What was most valuable about learning about resources in your community?
   2. [If the platform did not help them learn about resources]: What could be improved to help you learn more about resources in your community?

**Referral Experience(s):**

1. At any point did you pursue requesting services using the platform?
   1. Did you receive any referrals to community organizations?
      1. If no: What types of services were you hoping to be matched with?
      2. If yes: 5b
   2. Did you make any requests for services yourself?
      1. If no: please tell me about why you didn’t pursue a self-referral
      2. [Yes, requested services]: Please tell me about your experience getting connected with the services you requested.
         1. How long did it take to hear back? Did use of the platform in any way facilitate your first interaction(s) with that organization?
      3. In what ways could we improve this process?
   3. Were you at any point referred to a program **by an organization** to other available services?
      1. Were these services provided within the community at large or platform?
      2. How, if at all, did this referral experience differ from your self-initiated referrals?
2. Are there services that you would like to have available in the platform?
   1. Do you feel you had needs that were not met?

**Overall experience and Future Plans:**

1. What did you like most or find most useful about using the platform?
   1. Were there any resources that you didn’t see on the platform that should be available?
2. Were there any aspects of using this app that you found challenging?
   1. [If yes]: What were the most challenging aspects?
   2. Are there ways we can make the platform easier to use moving forward?
3. Do you view this platform as a useful resource for members of the cancer community?
4. A possible long-term vision for this platform is to be connected with local health care systems and health care providers, with whom patients could opt to share information. What information, if any, would you want to be shared with your care team or health care provider(s)?
5. Is there anything we missed that you would like to add?

*Closing remarks: Thank you for participating. Your input will help us to successfully develop this new mobile application to connect people with helpful social and community resources and services.*

*We will be continuing to develop the platform beginning in January–would you be interested in participating in helping us design the platform?*

*As a way to thank you for your time, $25 will be added to your ClinCard within the next few days.*

# Part 4: Participating CBOs

| **Populations Served** | | **CBO Organization Description** |
| --- | --- | --- |
| All cancers | Patients and survivors. | Offers 8 free one-on-one sessions with a qualified personal trainer. Our mission is to help people who have cancer, or who have had cancer, become as healthy as possible through strength training and other physical activity. |
| All cancers | Patients, survivors, and families. | Provides free social and emotional support for anyone impacted by any type of cancer, including people living with cancer, and their families, friends, and caregivers. Our comprehensive program is built on five pillars of support: support groups, healthy lifestyle classes, educational opportunities, social connections, and resources & referrals—all designed to ensure that no one faces cancer alone. |
| Specific cancer | Patients, survivors, and families. | A statewide nonprofit organization serving those affected by ovarian cancer from throughout the Upper Midwest. The CBO is dedicated to funding research, providing support to women and families, educating the medical community and raising awareness.” |
| All cancers | Patients in active treatment, families, and caregivers. | CBO’s mission is to offer relief to cancer patients and their loved ones through financial assistance, education, and emotional support. |
| All cancers | Patients and survivors. | A cancer thriver-led organization offers compassionate, holistic support, including expert guidance and emotional support to individuals and families navigating the cancer journey. Always Navigating Your Cancer Journey TOGETHER. |
| All cancers | Patients and survivors. | CBO engages the law to resolve complex challenges facing people and communities affected by cancer. Our mission and values exist in service to the vision we hold for a world that embraces equity and justice as fundamental to health.” |
| Specific cancer | Patients, survivors, and families. | CBO was founded by a group of lung cancer patients who were concerned about the lack of awareness and research funding for lung cancer, the deadliest of all cancers. Raising awareness about lung cancer creates a path toward improved wellness, earlier detection, and an increased survival rate. This CBO funds lung cancer research, provides support for lung cancer patients, and educates the community. |
| Specific cancer | Patients, survivors, families, and at-risk populations. | CBO core mission is to end colorectal cancer deaths by increasing screening and educating others about the signs and symptoms of this preventable disease. |
| All cancers | Caregivers and families. | CBO’s mission is to change the way caregivers of all kinds are seen and supported. They provide direct support to caregivers through podcasting, support groups, social media content, coaching, and caregiver gift boxes. In addition, they provide education and tools to those in a position to support caregivers such as medical students and professionals, faith leaders, and community members. |
| All cancers and other illnesses. | Patients and their families/caregivers. | CBO provides medically tailored meals curated by registered dietitians and trained chefs for patients with cancer and other illnesses. It also provides nutrition counseling and education services. |
| Specific cancer | Patients and survivors | CBO provides free, personalized one-to-one support for women who have been impacted by a breast cancer diagnosis. Our unique mentoring program extends the patients’ support network beyond their family and medical team—we connect them with a Peer Mentor Guide who shares a similar experience and lifestyle. |
| All cancers | Patients and their families/caregivers. | CBO offers a home away from home for people facing cancer and their caregivers when cancer treatment is far away. |
| All cancers and other illnesses. | Families and caregivers. | CBO provides free resources and services to all children, young adults, and adults who are grieving the death or terminal diagnosis of a beloved family member. Our model of care is truly exceptional, providing one-of-a-kind support logistically, psychosocially, and financially. We realize each family member grieves differently and, therefore, has different needs. |
